# Ruptured Intracranial Aneurysm in a 60-Day-Old Infant: An Extreme Case

**DOI:** 10.7759/cureus.53442

**Published:** 2024-02-02

**Authors:** Regina Pinto Silva, Cláudia Teles Silva, Marta João Silva, Augusto Ribeiro

**Affiliations:** 1 Pediatrics, Centro Hospitalar Universitário de São João, Porto, PRT; 2 Pediatric Intensive Care Unit, Centro Hospitalar Universitário de São João, Porto, PRT

**Keywords:** surgical treatment, endovascular treatment, dissecting etiology, infant, cerebral aneurysm

## Abstract

The prevalence of aneurysms in children is low when compared to adults, being even rarer in the first year of life. They can be secondary to infections, traumatic brain injury, autoimmune diseases, or connective tissue diseases. Dissecting etiology is rare. A 60-day-old female infant, previously healthy, presented to the emergency department (ED) with irritability and loss of appetite since the preceding day, a fever of one-hour duration, and vomiting. Laboratory analysis revealed a hemoglobin level of 6.5 g/dL, without elevation of inflammatory markers. In the ED, she experienced two episodes, with a one-hour interval, of clonic movements of the upper eyelid and right upper limb, along with conjugate gaze deviation to the same side, which resolved after intravenous diazepam. Levetiracetam was initiated after the second episode. The anterior fontanelle became progressively tense. Brain computed tomography (CT) showed a voluminous intraparenchymal and subarachnoid hemorrhage with an aneurysm at the bifurcation of the left middle cerebral artery (MCA). Initially, an endovascular approach was tried but was not successful due to technical problems. Consequently, a Vaso-CT scan was performed that confirmed a dissecting aneurysm/pseudoaneurysm (8 mm × 10 mm × 10 mm) of the left MCA, originating from the upper wall of the M1 segment. Next, she underwent microsurgical exclusion of the aneurysm using microclips. Post-surgery brain CT showed acute ischemia in the entire MCA region. Follow-up angiography showed complete exclusion of the aneurysm. She evolved to grade 3 monoparesis of the upper limb at the six-month interval follow-up, which has been gradually improving with physical rehabilitation. The next-generation sequencing (NGS) panel for aneurysms and arterial dissections did not detect any pathogenic variants. Clinical presentation of cerebral aneurysms in infants can be subtle, and a high index of suspicion is required in cases of irritability, altered consciousness, seizures, bulging fontanelle, and motor deficits. Early detection is of utmost importance as it is associated with moderate mortality. Surgical treatment with the use of clips proved to be effective in this case.

## Introduction

This article was previously presented as a meeting abstract and poster at the 2023 Excellence in Pediatrics Conference held on December 1, 2023.

The prevalence of aneurysms in the pediatric age group is low compared to adults. It is even rarer in the first year of life. In the pediatric population, they seem more frequent in males than females (1,8:1) [1].

A cerebral aneurysm is a localized, abnormal dilation or ballooning of a blood vessel within the brain. These vascular anomalies can have significant clinical implications, ranging from asymptomatic incidental findings to life-threatening ruptures (10-23% of mortality). More frequently, they occur in the carotid bifurcation and posterior circulation, whereas large and giant aneurysms (larger than 2.5 cm in diameter) and related spontaneous thromboses are more common. In children, 20% are classified as a giant aneurysm; in this situation, it is four times more likely to present with subarachnoid hemorrhage [2].

In the pediatric population, they often pose distinct clinical and anatomical characteristics that require a tailored approach. Unlike adult aneurysms, which are more frequently associated with risk factors such as hypertension and atherosclerosis, pediatric aneurysms tend to occur due to an underlying predisposing disorder, such as traumatic brain injury, sickle cell anemia, cardiovascular diseases, autoimmune diseases, immunodeficiencies, connective tissue diseases, dysmorphic syndromes, and positive family history [3].

In the realm of pediatric aneurysms, the occurrence of spontaneous dissecting cases stands out as an unusual contrast to traumatic aneurysms. These cases, constituting 7% of all dissections in pediatric patients, play a significant role in triggering ischemic strokes. Notably, infants experience arterial dissections more commonly in the carotid artery territory, particularly affecting the internal carotid artery (ICA) and middle cerebral artery (MCA). It’s essential to highlight the rarity of arterial dissection in the posterior intracranial circulation in this specific demographic [4].

The clinical presentation is explained by the subarachnoid and intraparenchymal hemorrhage and the associated mass effect. This can present as headaches, seizures, motor-sensory deficit, or even death [5]. The diagnosis is made by brain computed tomography (CT) angiography, cerebral magnetic resonance, and the gold standard angiography [6]. Nowadays, there are two types of treatment: endovascular and microsurgical. Many studies have been made, but until this date, there is no evidence of superiority between them [7].

We present a case of a spontaneous dissecting aneurysm in a 60-day-old female infant.

## Case presentation

A two-month-old female infant was brought to the emergency department (ED) with irritability and less appetite since the preceding day, a fever of one-hour duration, and vomiting. Notably, there was no reported history of trauma or infection. The infant had no previous exposure to medication, and her medical records showed no pertinent medical issues. Furthermore, there was no familial background indicating congenital neurological or vascular diseases. As the patient was born prematurely at 36 gestational weeks through normal delivery, the perinatal period was uneventful, and the infant achieved typical growth and developmental milestones.

At her initial examination, she had a preserved global state and normal vital signs. The strength of the four limbs was preserved, and her pupils were symmetrical and reactive to the light. 

Laboratory tests showed anemia with a hemoglobin level of 6.5 g/dL, without elevation of inflammatory parameters. Urinalysis excluded urinary tract infection. Blood and urine cultures were negative. A blood transfusion was carried out due to the anemia, which had a good response. 

In the ED, she experienced two episodes, with a one-hour interval, of clonic movements of the upper eyelid and right upper limb, along with conjugate gaze deviation to the same side, which resolved after intravenous diazepam. Levetiracetam was initiated after the second episode.

**Figure 1:**
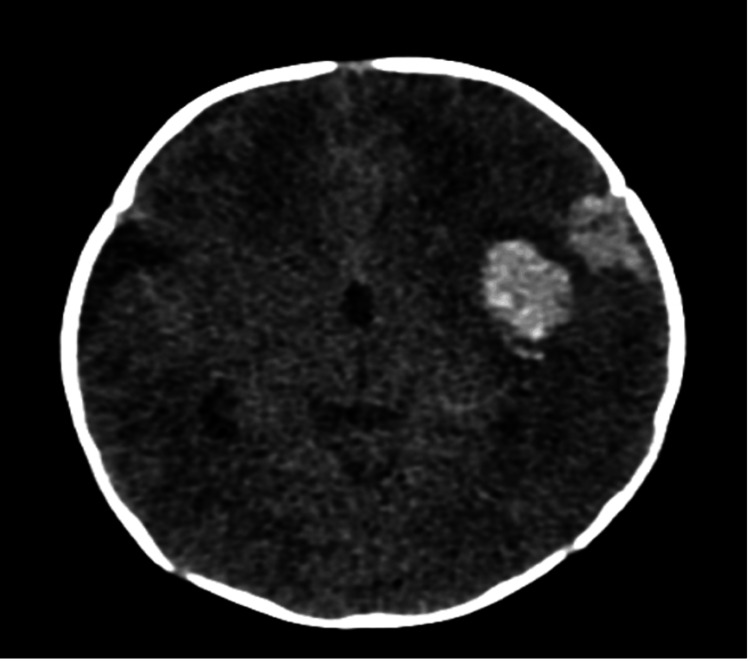
Admission brain computed tomography (CT) scan showing a voluminous intraparenchymal hemorrhage

Clinically, she started to present a bulging anterior fontanelle, without anisocoria or motor deficits. A brain CT scan showed a voluminous intraparenchymal and subarachnoid hemorrhage with an aneurysm at the bifurcation of the left MCA (Figure 1). 

The patient was admitted to the pediatric intensive care unit, where she was sedated with midazolam, fentanyl, and rocuronium and intubated. Neuroprotection strategies were carried out. She has had no more clinical seizures since the start of midazolam perfusion. 

**Figure 2:**
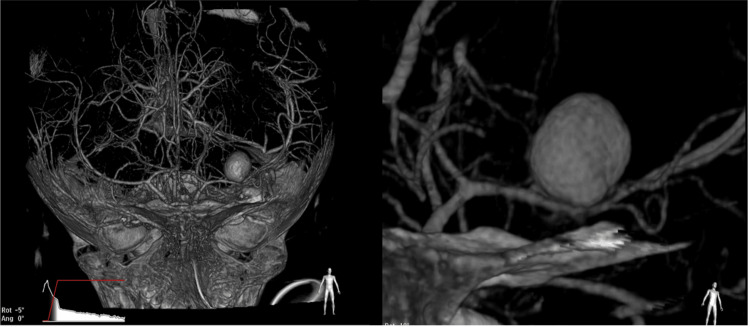
A Vaso-CT scan Right: a dissecting aneurysm/pseudoaneurysm (8 mm × 10 mm × 10 mm) of the left middle cerebral artery, originating from the upper wall of the M1 segment. Left: the same aneurysm amplified.

Initially, an endovascular approach was tried. However, this procedure was not successful due to technical problems. Consequently, a Vaso-CT scan was performed that confirmed a dissecting aneurysm/pseudoaneurysm (8 mm × 10 mm × 10 mm) of the left MCA, originating from the upper wall of the M1 segment (Figure 2).

Next, she was submitted to emergency neurosurgery under general anesthesia with the successful exclusion of the brain aneurysm with microclips. The dissecting etiology was confirmed intraoperatively. No complications happened during the procedure. 

During her hospitalization in the pediatric intensive care unit, the patient underwent three Doppler studies of the brain and neck arteries, all of which revealed the absence of vasospasm. Initial electroencephalography (EEG) demonstrated epileptic activity localized to the left frontal lobe, a finding that was not replicated in an EEG conducted three weeks post-event. Importantly, the patient did not experience any further clinical seizures following surgery. Nevertheless, she is still medicated with levetiracetam, which was initiated on the second day of admission to the pediatric intensive care unit. She is currently undergoing tapering, as she is not experiencing seizures.

Sedation was weaned, and she was successfully extubated on the 10th day. Neurologically, after the withdrawal of sedation, a diminished strength of the upper right arm was noticed, with no other neurological findings. On day 16, she was transferred to the pediatric ward. 

**Figure 3:**
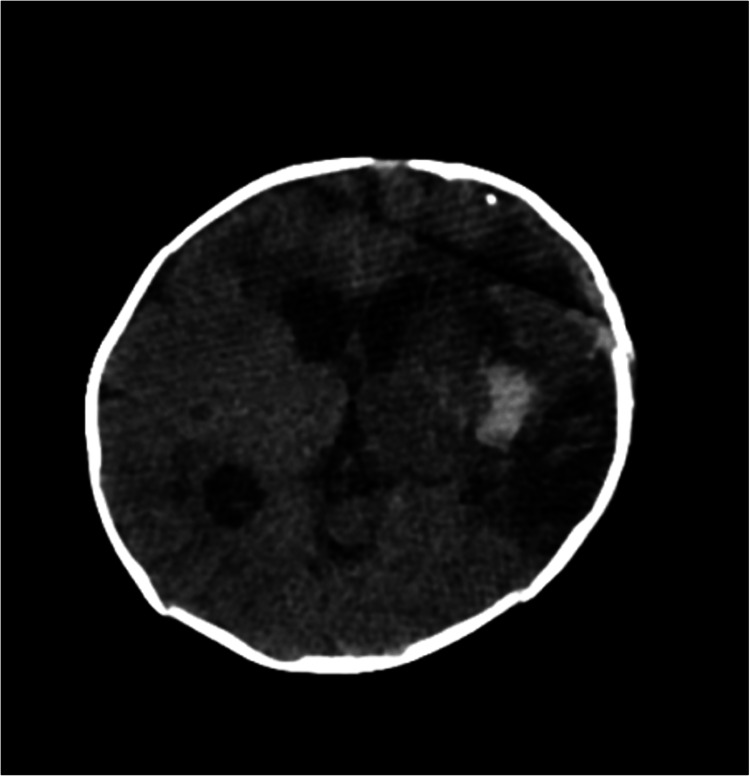
Post-surgery brain computed tomography (CT) showing partial reabsorption of the intraparenchymal and subarachnoid hemorrhage and recent ischemia in the entire middle cerebral artery region

**Figure 4:**
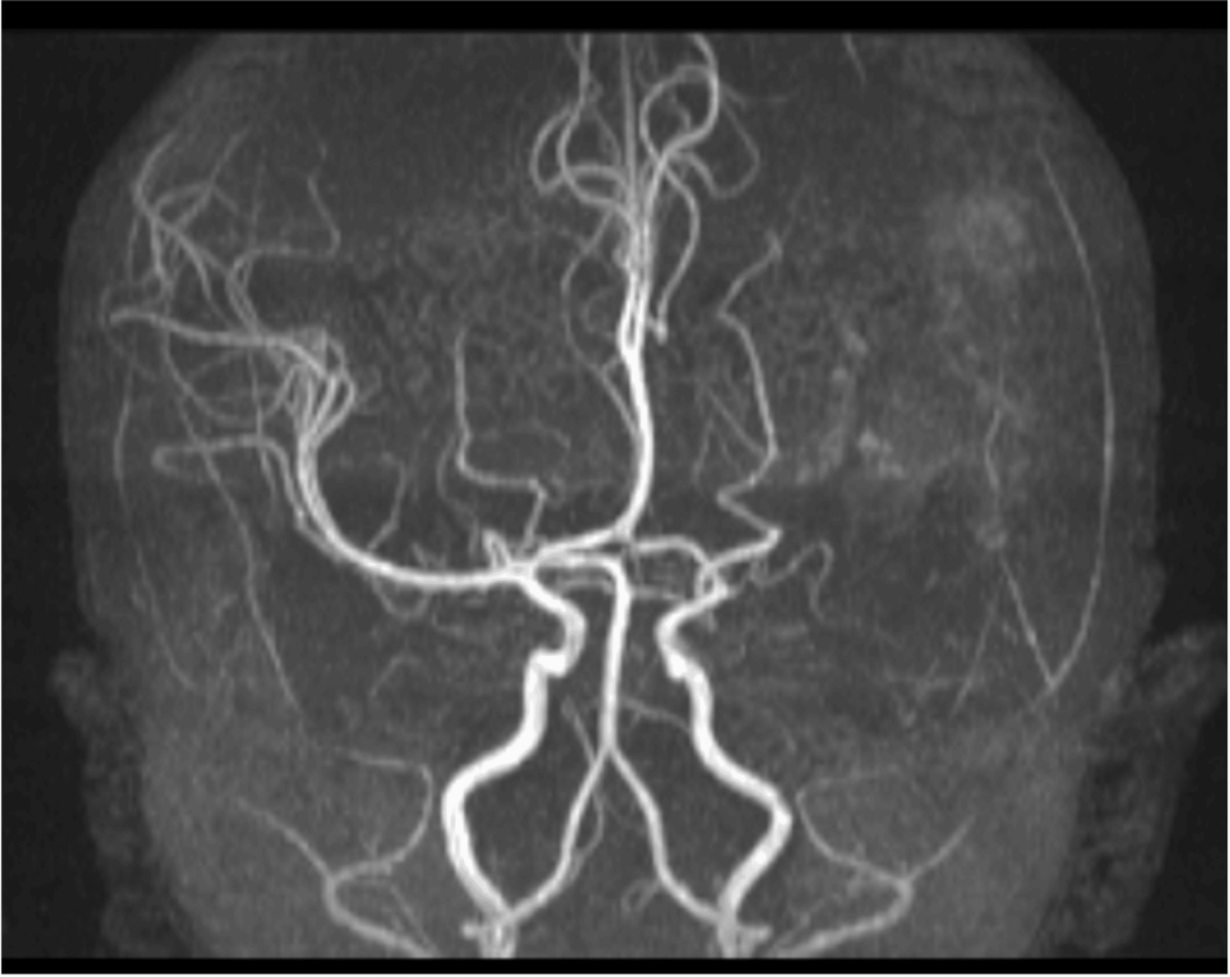
Magnetic resonance angiography showing the exclusion of the aneurysm and no other vascular malformation

One week post-surgery, a follow-up brain CT scan indicated a reduction in the volume of the hemorrhage and recent ischemia in the entire MCA region (Figure 3). Three weeks later, magnetic resonance angiography (MRA) was performed, revealing the successful exclusion of the aneurysm and no presence of other vascular malformations (Figure 4). An additional imaging study was conducted to screen for aneurysms in other locations, particularly at the abdominal level, and it yielded normal results. The next-generation sequencing (NGS) panel for aneurysms and arterial dissections did not detect any pathogenic variants.

After a hospital stay of 34 days, the infant was discharged with a good overall health status. However, she evolved to grade 3 monoparesis of the upper limb at the six-month interval, which has been gradually improving with physical rehabilitation. She maintains hospital follow-ups in neurology, neurosurgery, genetics, and rehabilitation medicine.

## Discussion

We report the case of a two-month-old female infant with a left MCA aneurysm due to spontaneous dissection, in whom the aneurysm was obliterated with the use of microclips through surgery. 

In the pediatric population, the incidence of intracranial aneurysms (IAs) is rare, yet their impact is markedly more severe than in adults. Hemorrhage constitutes the predominant manifestation in about two-thirds of cases, showcasing a rebleeding rate that surpasses that observed in the adult population [1].

The origin of pediatric aneurysms is primarily linked to pre-existing predisposing conditions like infection, tumor, trauma, or dissection. Furthermore, specific genetic disorders, such as Marfan syndrome, moyamoya disease, and Ehlers-Danlos syndrome, have been identified as contributors to this condition [4].

Infantile aneurysms have traditionally seen a high incidence of traumatic cases, with brain trauma from either injury or surgical procedures contributing to around 40% of all pediatric aneurysms. Conversely, the occurrence of spontaneous dissecting aneurysms remains a rare phenomenon in the pediatric demographic [7].

As previously stated, the clinical manifestations of an aneurysm can be due to the mass effect. In our case, the infant presented with non-specific symptoms such as fever, irritability, vomiting, and food refusal, which initially made the diagnosis challenging. It’s noteworthy that there was no history of trauma or infection, and the patient had an unremarkable birth history. Diagnosing a dissecting MCA aneurysm in an infant is complicated due to the lack of specific clinical signs and symptoms. The initial presentation with fever and non-focal neurological signs did not immediately raise suspicion of an intracranial vascular abnormality. It was only after the onset of seizures and a thorough evaluation, including brain imaging and venous-CT, that a brain aneurysm was diagnosed, and a dissecting etiology was found.

The ultimate goal of neurovascular intervention is to reduce the risk of aneurysmal hemorrhage, reduce the mass effect, and preserve cerebral vasculature. Treatment options for cerebral aneurysms encompass endovascular coiling and surgical clipping. The decision between the two relies on the patient’s symptoms and age, with a growing preference for endovascular coiling due to its lower mortality rates and shorter hospital stays in contrast to clip placement. Notably, there is a scarcity of comprehensive outcome data for these treatments in pediatric cases. Within the realm of microsurgical interventions, there are several options, including clip reconstruction, trapping, or bypass with proximal occlusion. Notably, the choice of surgically obliterating the aneurysm sac has shown an association with decreased recurrence rates and a subsequent reduction in the development of de novo aneurysms [8,9].

Treatment options for cerebral aneurysms encompass endovascular coiling and surgical clipping. The decision between the two relies on the patient’s symptoms and age, with a growing preference for endovascular coiling due to its lower mortality rates and shorter hospital stays in contrast to clip placement. Notably, there is a scarcity of comprehensive outcome data for these treatments in pediatric cases.

In our patient, we tried the endovascular treatment first. However, due to complications, a surgical treatment had to be pursued. Fortunately, the surgical procedure was successful without complications. However, postoperatively, the patient did exhibit reduced strength in the right upper arm, emphasizing the need for ongoing rehabilitation and close follow-up care.

One of the intriguing aspects of this case was the transient epileptic activity detected in the left frontal lobe, which resolved after surgery. This highlights the potential impact of aneurysms on adjacent brain tissue and the importance of monitoring for neurological complications.

The long-term follow-up of this infant revealed positive results, with the exclusion of the aneurysm observed on MRA and no pathological genetic variants detected. However, the residual motor deficit in the right upper arm underlines the importance of continued rehabilitation and neurological assessment in pediatric patients following complex neurosurgical procedures.

As seen in our case, positive outcomes are frequently attainable in the management of pediatric IAs when handled with careful consideration.

Nevertheless, this patient demographic remains prone to the emergence of de novo or enlarging aneurysms, underscoring the necessity for prolonged surveillance [10]. In the pediatric population, opting for MRA is a common strategy for surveillance imaging to mitigate exposure to excessive ionizing radiation. Despite its prevalent use, MRA is not without limitations, including a diminished ability to detect aneurysms with slow flow, challenges in visualizing those smaller than 3 mm, and an extended image acquisition time that may require the use of anesthesia, especially in young children. Until a more advanced and logistically convenient modality emerges, MRA continues to serve as a practical choice for these patients in the interim [11,12].

## Conclusions

In conclusion, cerebral aneurysms are rare during the first year of life, especially those with a dissecting etiology. Our case of a dissecting MCA aneurysm in a two-month-old infant underscores the need for a high index of suspicion and comprehensive evaluation when infants present with non-specific symptoms as clinical presentation can be subtle, so a high index of suspicion is required in cases of irritability, altered consciousness, seizures, bulging fontanelle, and motor deficits.

Early recognition, prompt diagnosis, and expert multidisciplinary management, including neurosurgery, neurology, and rehabilitation, are vital for achieving favorable outcomes. This case also highlights the potential for transient neurological complications in such cases, underscoring the importance of vigilant postoperative care and follow-up in pediatric patients with IAs. Further research and case studies are warranted to better understand the pathophysiology, optimal diagnostic strategies, and treatment approaches for this rare condition in infants.
