# Correction: Ruptured Intracranial Aneurysm in a 60-Day-Old Infant: An Extreme Case

**DOI:** 10.7759/cureus.c187

**Published:** 2024-08-01

**Authors:** Regina Pinto Silva, Cláudia Teles Silva, Marta João Silva, Augusto Ribeiro

**Affiliations:** 1 Pediatrics, Centro Hospitalar Universitário de São João, Porto, PRT; 2 Pediatric Intensive Care Unit, Centro Hospitalar Universitário de São João, Porto, PRT

This article has been corrected to remove Pedro Alberto Silva from the author list. Dr. Silva was erroneously included in the author list despite not contributing to the work. The authors regret that this error was not identified prior to publication.

